# Classification of diabetic retinopathy: Past, present and future

**DOI:** 10.3389/fendo.2022.1079217

**Published:** 2022-12-16

**Authors:** Zhengwei Yang, Tien-En Tan, Yan Shao, Tien Yin Wong, Xiaorong Li

**Affiliations:** ^1^ Tianjin Key Laboratory of Retinal Functions and Diseases, Tianjin Branch of National Clinical Research Center for Ocular Disease, Eye Institute and School of Optometry, Tianjin Medical University Eye Hospital, Tianjin, China; ^2^ Singapore Eye Research Institute, Singapore National Eye Centre, Singapore, Singapore; ^3^ Duke-National University of Singapore Medical School, Singapore, Singapore; ^4^ Tsinghua Medicine, Tsinghua University, Beijing, China

**Keywords:** diabetic retinopathy, classification, severity staging system, pathophysiology, imaging technology, artificial intelligence, deep learning, quantitative assessment

## Abstract

Diabetic retinopathy (DR) is a leading cause of visual impairment and blindness worldwide. Since DR was first recognized as an important complication of diabetes, there have been many attempts to accurately classify the severity and stages of disease. These historical classification systems evolved as understanding of disease pathophysiology improved, methods of imaging and assessing DR changed, and effective treatments were developed. Current DR classification systems are effective, and have been the basis of major research trials and clinical management guidelines for decades. However, with further new developments such as recognition of diabetic retinal neurodegeneration, new imaging platforms such as optical coherence tomography and ultra wide-field retinal imaging, artificial intelligence and new treatments, our current classification systems have significant limitations that need to be addressed. In this paper, we provide a historical review of different classification systems for DR, and discuss the limitations of our current classification systems in the context of new developments. We also review the implications of new developments in the field, to see how they might feature in a future, updated classification.

## Introduction

Diabetes mellitus (DM) is one of the fastest growing chronic diseases in terms of global prevalence (1). According to recent data published by the International Diabetes Federation, approximately 537 million adults had diabetes in 2021, while estimates suggest that this figure will increase to 783 million by 2045 (2). Diabetic retinopathy (DR) is an important microvascular complication, and occurs in about 30% of individuals with diabetes (3, 4). DR is therefore a leading cause of preventable vision impairment and blindness among adults, particularly in higher-income countries (5). With the overall incidence of diabetes rapidly increasing, the number of adults worldwide with DR, vision-threatening DR, and diabetic macular edema (DME) are projected to increase to approximately 161 million, 45 million, and 29 million, respectively by 2045 (6).

Since the first description of retinal changes in diabetes, the emphasis has predominantly been on vascular abnormalities in DR. This is not surprising, as the early ophthalmoscopically-visible lesions in DR, such as intraretinal hemorrhages, venous abnormalities, lipid exudates and other changes, primarily reflect retinal capillary abnormalities, which has been confirmed on histopathological studies (7, 8). Eventually, these vascular abnormalities and retinal ischemia result in diabetic macular edema (DME) and retinal vasoproliferative complications, which can lead to vision loss and blindness. Over decades, various DR staging and classification systems have sought to accurately describe the progression of DR, quantify severity of the disease, and stratify risk of progression. Early classifications from the mid-20^th^ century, such as the ophthalmoscopic classification (9) and Hammersmith grading system (10) have been abandoned as our understanding of the disease has improved. More recently, the Early Treatment of Diabetic Retinopathy Study (ETDRS) classification (11) has been considered the “gold standard” for many years, because it was developed and validated on natural history data that demonstrated its ability to prognosticate risk of progression to proliferative disease and vision loss (12). The ETDRS classification is still used for research and clinical trials today, but its widespread clinical application is limited by its complexity. In everyday clinical practice, the International Clinical Diabetic Retinopathy (ICDR) Severity Scale (13), which in essence is a simplified ETDRS system, is currently the most commonly used classification system worldwide. Previous classification systems had to be updated or replaced as our understanding of the disease improved. In the years since the adoption of the ETDRS and ICDR staging systems, there have been major developments, including better understanding of the pathophysiology of DR, recognition of retinal neural dysfunction and neurodegeneration, improvements in imaging technology, and the development of disease modifying treatments, such as anti-vascular endothelial growth factor (anti-VEGF) therapy. Considering these massive strides that have been made in the field, we feel that it is timely to review the progress made, and determine if it is time for an update to our existing classification systems for DR.

Therefore, we aim to provide a historical review of different classification systems for DR, as well as to discuss the limitations of current classification systems in the context of new developments. We also review the implications of technological developments and new treatments for DR, to see how they might feature in an updated classification.

## Past: A historical review of classification systems for diabetic retinopathy

### Early classifications of DR

Diabetic retinal lesions such as hemorrhages and exudates were first observed by Eduard Jaeger using the direct ophthalmoscope in 1856 (14). However, there was limited evidence of a causal relationship between diabetes mellitus and retinopathy at the time, and many prominent ophthalmologists, such as Albrecht von Graefe, questioned the link (15). In the years that followed, more evidence linking diabetes and retinal complications began to emerge, including reports by Louis Desmarres in 1858 (16) and Henry Noyes in 1869 (17). In 1872, Edward Nettleship published a histopathological study demonstrating “cystoid degeneration of the macula” in diabetes (18). In 1876, German ophthalmologist Wilhelm Manz described fibrovascular proliferations along the blood vessels in a patient with proliferative diabetic retinopathy, which he termed “retinitis proliferans” at the time (19). Julius Hirschberg proposed the first classification of DR in 1890, which he sub-divided into 3 types: retinitis centralis punctata (which affected mainly the posterior pole), retinitis hemorrhagica, and other retinal manifestations (20). “Diabetic retinitis” was a frequently used term at the time, because it was presumed that exudation was related to inflammation. In 1934, Wagener, Dry and Wilder proposed an expanded classification which included 5 stages and incorporated lesions such as hemorrhages, punctate exudates, cotton-wool exudates and venous changes, with proliferative retinopathy being the most severe stage of disease (21). Subsequently in the 1940s, Arthur James Ballantyne described capillary wall alterations and microaneurysms in DR, and included them in a classification of DR (22).

As DR was studied in greater depth, more classification systems for DR were proposed over the next decades. In the early 1950s, Scott suggested a six-stage clinical classification of DR (23). In stages I to III, various lesions that we now understand as pre-proliferative disease were described, including capillary microaneurysms, intraretinal hemorrhages, exudates and venous changes. At the time, it was not recognized that vitreous hemorrhage was a direct consequence of neovascularization, and so vitreous hemorrhage was classified as a separate stage IV, which was thought to subsequently progress to proliferative disease. Stage V was “retinitis proliferans”, which was subdivided into V(a), retinitis proliferans, and V(b), the “vascular type” of retinitis proliferans, while stage VI was retinal detachment and “gross degenerative changes”, representing end-stage diabetic retinal disease. One of the major drawbacks of this classification system was the fact that the pre-proliferative stages of disease were still divided primarily by specific lesion type – for example, the presence of exudates necessitated classification as stage III, whereas we now know that the development of hard exudates or macular edema can progress independently of overall retinopathy status.

### Grading of individual lesions

In 1966, Lee et al. proposed an updated DR classification system, which started to resemble more modern classification systems. Recognizing that different specific lesion types (such as venous changes, microaneurysms and hemorrhages, exudates) do not necessarily progress together, but can vary in terms of severity, they proposed grading each of these lesions types on individual severity scales (9). Based on detailed examination with binocular indirect ophthalmoscopy, and detailed fundus drawings from 400 patients with DR, they proposed individual 5-point severity gradings for each of four lesion types: 1. angiopathy (with separate sub-gradings for A. venous dilatation, B. microaneurysms & hemorrhages, and C. neovascularization), 2. exudates, 3. proliferative retinopathy, and 4. vitreous hemorrhage. After individual lesion classification, they then looked at the eye as a whole to determine which type of retinopathy predominated. Astutely, they also included separate classification for additional “Other Changes”, including macular changes, rubeosis iridis and secondary glaucoma, retinal detachment, and optic nerve changes. Naturally, one of the major drawbacks to this classification system was that it relied on ophthalmoscopy and detailed fundus drawings, which were time-consuming and prone to inter-observer variability.

### Photographic classification: Hammersmith grading system

As reproducibility and consistency were clearly important for a universal DR staging system, the introduction of fundus photography into the classification systems represented a major breakthrough. Fundus drawings from indirect ophthalmoscopy were an important method for recording the appearance of the retina and the progress of visible lesions for many years. However, as assessments of DR and individual lesion severity relied on more objective assessment of lesion size, extent, and number, this approach became increasingly impractical. Fundus photographs were more objective, and could even be used to evaluate progression of DR severity in the same patient at different time points. In 1967, the Hammersmith grading system was the first to describe the severity of DR by using the fundus photographs (10). Five components of retinopathy such as microaneurysms and hemorrhages, exudates, new vessels, venous irregularities and retinitis proliferans, were recorded through four standard photographs. The Hammersmith grading system was widely used to document the changes in eyes associated with treatment (24). For example, in a study examining the effect of laser photocoagulation on proliferative DR in 90 eyes of 72 patients, severity grading by color fundus photography (CFP) was performed prior to laser treatment, and following laser treatment at yearly intervals (25). This allowed by objective evaluation of the effect of treatment, and analysis by the number of quadrants affected, It was also significant that in this study they acknowledged and included some patients with neovascularization outside the photographic fields of the Hammersmith grading system, which did highlight a drawback of the system at the time. Other examples of the Hammersmith photographic grading system in use included a large study involving 6792 diabetic patients in South India (26). This large cohort underwent clinical examination and fundus photography, graded according to the Hammersmith grading system. This allowed for estimation of the prevalence rates of DR in a large South Indian cohort.

### Airlie house classification

In 1968, over 50 experts from around the world met in the Airlie House, Virginia, USA to analyze current understanding of DR natural history, and to develop a standardized classification for DR (27). This was a major milestone in the classification and staging of DR. Some key elements in the natural history of DR that were identified and described include: “capillary occlusion is an essential early change prior to the formation of arteriovenous shunts in DR, which was contrary to previous popular cognition; newly formed blood vessels undergo a cycle of proliferation and degeneration; and vision will be seriously threatened when fibrous tissue or vitreous attached to the neovascularization shrinks” (28). The Airlie House classification that was produced, on which all our current modern DR classification systems are based, emphasizes a fundamental dichotomy of retinopathy between non-proliferative DR (NPDR) and proliferative DR (PDR). NPDR included various signs such as microaneurysms, hard and/or soft exudates, venous caliber abnormalities, venous sheathing, perivenous exudate, arteriolar abnormalities, intraretinal microvascular abnormalities (IRMAs), and arteriovenous nicking. PDR included retinal or disc neovascularization, fibrous proliferation, retinal detachment, preretinal and vitreous hemorrhage. This classification system relied on standardized 7-field stereoscopic CFP images, which were compared against a set of 18 standard color photographs.

### Modified airlie house classifications – the ETDRS severity scale

Modern DR classification systems that are in use today are largely based on the original Airlie House classification, and are frequently referred to as “modified Airlie House classifications”. Minor modifications were made to the Airlie House classification, for application in the Diabetic Retinopathy Study (DRS) (29) and Early Treatment of Diabetic Retinopathy Study (ETDRS) (30) in 1981 and 1991, respectively. Modifications that were made for the DRS classification include: assessment of location, extent, and severity of retinal thickening of macular edema; assessment of five features including hard exudates, soft exudates, arteriovenous nicking, retinal elevation, and vitreous hemorrhage as an additional step for the grading; separating previously combined characteristics into venous abnormalities and arterial abnormalities and grading them individually; addition of some characteristics such as microaneurysms, drusen, hard exudate rings, papillary swelling, and subretinal hemorrhage (30). In the ETDRS classification, fundus lesions and characteristics, such as hemorrhages/microaneurysms (H/Mas), venous beading and loops, hard exudates, IRMAs and neovascularization, were graded individually from standard 7-field 30°C fundus photographs, and based on these individual lesion gradings, an overall retinopathy severity level was determined at the eye level, with 14 levels ranging from level 10 (DR absent) to level 85 (advanced PDR, with posterior fundus obscured, or center of macula detached), excluding level 90 (for ungradable images) (12).

Another key contribution of the ETDRS clinical trials, was that they defined “clinically significant macular edema” (CSME). CSME was observed by using stereoscopic fundus photographs on the basis of the presence of retinal thickening and hard exudate (31), and was defined as: (a) Thickening of the retina at or within 500 µm of the center of the macula; or (b) hard exudates at or within 500 µm of the center of the macula, when associated with adjacent retinal thickening; or (c) a zone or zones of retinal thickening 1 disc area or larger, any part of which was within 1 disc diameter of the center of the macula (32). CSME was a crucial definition that influenced clinical management at the time, as the ETDRS trial established the therapeutic benefit of focal/grid laser photocoagulation for DME meeting the criteria for CSME (32).

Since its introduction in the early 1990s, the ETDRS severity scale has been the gold standard DR classification for both clinical and research clinical trial use. This is because the ETDRS study rigorously validated the severity scale, and demonstrated its prognostic value in predicting risk of progression to PDR, at 1-, 3- and 5-years, in a longitudinal cohort of 3,711 untreated eyes (12). This severity scale has been used in countless clinical and epidemiologic studies of DR over the past few decades, and has been an instrumental factor in improving our understanding and management of DR. One major drawback of this classification though, lies in its complexity. Because it requires detailed grading, it is frequently employed in research studies that have dedicated reading centers for standardized grading, but it is impractical for daily clinical use by ophthalmologists.

### The wisconsin epidemiologic study of diabetic retinopathy (WESDR)

One alternative classification system that attempted to overcome the issue of complexity with the ETDRS was proposed by the Wisconsin Epidemiologic Study of Diabetic Retinopathy (WESDR) study group. The WESDR was a population-based longitudinal cohort study that was started in 11 counties in southern Wisconsin from 1979 to 1980 (33–35). This cohort study included 1210 young patients with diabetes (age < 30 years) and 1780 older persons with diabetes (age ≥ 30 years) between 1980 and 1982. Over the next few decades, this large cohort of diabetic patients were systemically assessed for DR, and associated risk factors (36–45). DR was evaluated in a standardized manner by masked grading of standard 7-field stereoscopic CFPs throughout the study (46), and this was proposed as a simpler, less cumbersome alternative to the ETDRS severity scale.

Using the ETDRS severity scale, a grader would have to individually evaluate 21 lesions in each of the photographic fields for each eye, and use a computer program based on these gradings to assign the eye one of 14 possible severity levels (excluding ungradable images). In contrast, with the WESDR system, a grader examined all 7 photographic fields as a whole, and assigned the eye a severity level based on the greatest level of retinopathy severity present in any field. There were also fewer retinopathy severity levels in the WESDR system, ranging from level 1 to 7. To validate this simplified classification scale, they graded 4,604 eyes with both the WESDR and ETDRS scales, and demonstrated acceptable agreement. The exact agreement between the two scales was 78.3%, and the WESDR showed interobserver agreement of 78.5%, and intraobserver agreement ranging from 84% to 90% (47).

### The international clinical diabetic retinopathy (ICDR) severity scale

The ETDRS severity scale has been further simplified into the International Clinical Diabetic Retinopathy (ICDR) severity scale, for widespread daily clinical use. The ICDR severity scale essentially distills the 14 severity levels of the ETDRS severity scale, into 5 levels of retinopathy severity. Because of its convenience and ease of adoption, the ICDR severity scale is by far the most common classification system in clinical use around the world.

The ICDR severity scale was developed from the ETDRS and WESDR data and classification systems, through an international consensus workshop in 2002. An initial planning meeting including representatives from five countries was held in conjunction with the Annual Meeting of the American Academy of Ophthalmology (AAO) in 2001. Thereafter, in 2002, 14 individuals from 11 countries attended the International Congress of Ophthalmology in Sydney and developed the ICDR through discussion and consensus *via* a modified Delphi system (13). This classification system was deliberately intended to be convenient and easy to use in everyday clinical practice by general ophthalmologists and primary care physicians. The ICDR severity scale, along with the corresponding ETDRS severity scale levels, are shown in **Table 1**. Various international clinical guidelines for DR management, such as the International Council of Ophthalmology (ICO) guidelines, use the ICDR severity scale for recommendations of management and follow-up surveillance intervals for DR (48).

**Table 1:** ICDR and corresponding ETDRS severity scale levels.

ICDR severity levels	ETDRS severity levels	
No apparent retinopathy	Level 10	No retinopathy
Mild NDPR	Level 20	Very mild NPDR
Moderate NPDR	Level 35	Mild NPDR
	Level 43	Moderate NPDR
	Level 47	Moderately severe NPDR
Sever NPDR	Level 53	Severe NPDR
PDR	Levels 60, 61	Mild PDR
	Level 65	Moderate PDR
	Levels 71, 75	High-risk PDR
	Levels 81, 85	Advanced PDR

ICDR, International Clinical Diabetic Retinopathy; ETDRS, Early Treatment of Diabetic Retinopathy; NPDR, non-proliferative diabetic retinopathy; PDR, proliferative diabetic retinopathy.

## Present: Limitations of current DR classification systems

Current DR classification systems are reproducible, well-validated, and are robust in prediction of important outcomes of clinical interest. However, major developments over the past few decades since their introduction have resulted in some important limitations.

First, the current DR classifications rely only on 7 standard field photographs to grade the severity of DR. However, these standard photographic fields only cover about 30% of the total retinal surface area (49). Peripheral retinal lesions may have important prognostic significance and may improve prediction of future clinical outcomes. Second, DME is now the most common cause of visual impairment from DR (50), and the presence of DME influences clinical management and treatment. however, our DR classification system prognosticates the risk of progression to PDR, and does not effectively predict the incidence of DME, nor does it adequately account for different levels of DME severity. Third, our classification systems do not take into account measures of visual function, such as best-corrected visual acuity, or other aspects of visual function, such as contrast sensitivity, visual quality, visual fields, low luminance acuity, and metamorphopsia. Inclusion of such outcome measures may be important as new therapies are developed. Beyond measures of visual function alone, patient-reported outcome measures and quality of life may also need to be taken into account.

Fourth, our DR classifications focus only on the vascular aspect of disease, and do not include evaluation of the neural retina or diabetic retinal neurodegeneration. There is evidence now that early neural degeneration may precede or accompany vascular lesions, and these changes may have impact on visual function (51, 52). Fifth, evaluation of systemic health is absent in current classifications, although it is clear that systemic factors such as diabetes duration, glycemic control, co-morbid hypertension and dyslipidemia, and even pregnancy, can influence DR progression and outcomes (3, 53, 54).

Sixth, current classification systems do not record the regression or resolution of retinal neovascularization. If the PDR scale was revised to describe the key levels, it could help to improve the characterization of the natural history of eyes with PDR and be an outcome measure for treatment. Seventh, the current classification system fails to address reductions of DR severity that are seen after intravitreal anti-VEGF treatment. It is currently unclear how improvements in DR severity level with such treatments modify the undertlying disease process, and affect future clinical outcomes. Finally, current DR severity scales and individual lesion gradings are not quantitative. Quantitative staging systems may facilitate research, and provide better prognostication (55). With these limitations in mind, it is clear that improvements and updates are needed to our existing DR classification systems.

## Future: New developments that will influence a new DR classification system

### Pathophysiologic mechanisms

**Figure 1:**
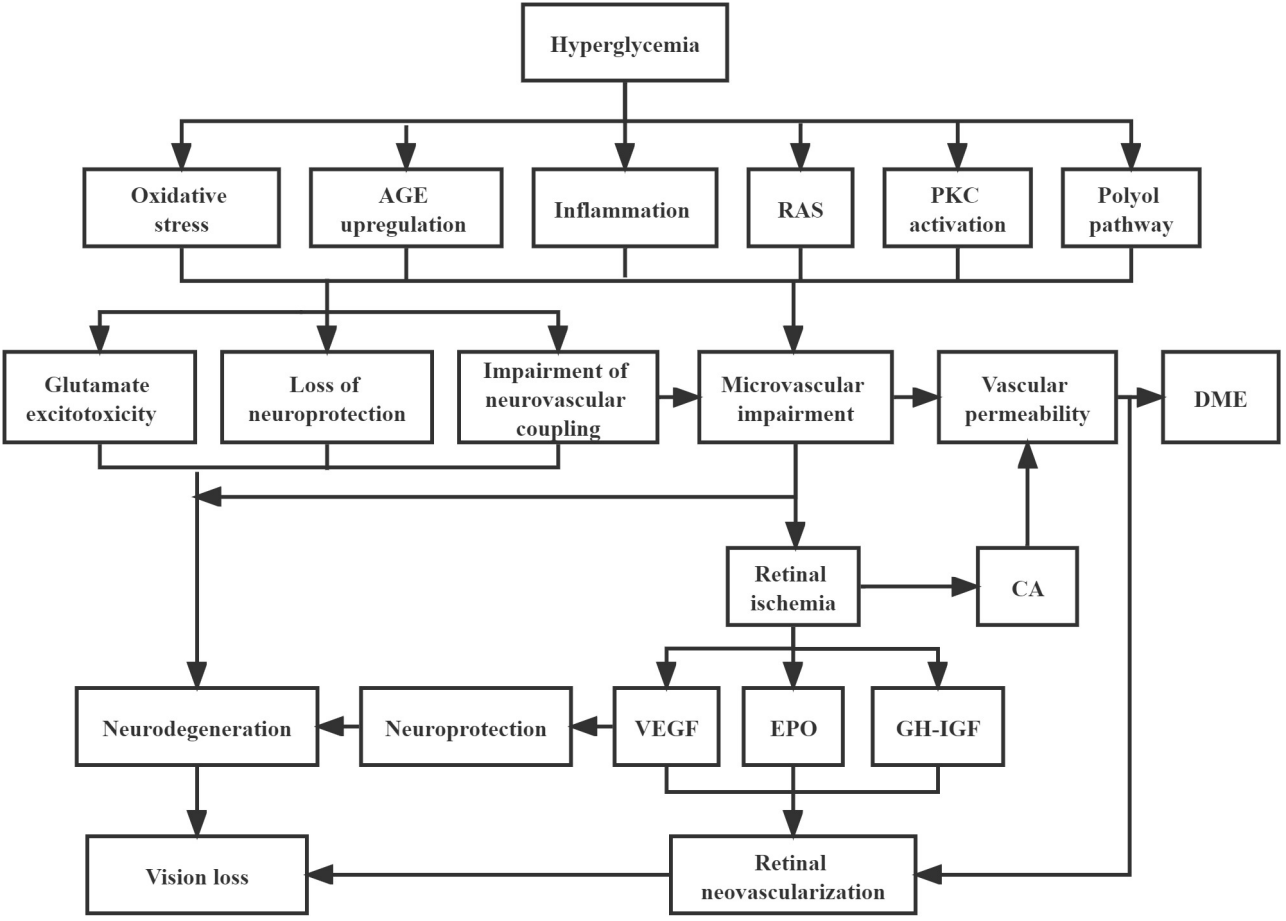
Pathophysiology of diabetic retinopathy Hyperglycaemia cascade of events leading to neurodegeneration and microvascular impairment, which are the two key main pathways to result in the development of diabetic retinopathy. Neurodegeneration can be activated by glutamate excitotoxicity, loss of neuroprotection, and impairment of neurovascular coupling. Meanwhile, impairment of neurovascular coupling can lead to microvascular impairment, which can trigger the formation of DME and retinal neovascularization. AGE, advanced glycation end-products; RAS, renin-angiotensin system; PKC, protein kinase C; DME, diabetic macular edema; CA, carbonic anhydrase; VEGF, vascular endothelial growth factor; EPO, erythropoietin; GH-IGF, growth hormone-insulin growth factor.

As we understand more about the pathophysiologic mechanisms that drive DR progression and its complications, this knowledge is likely to influence new classification systems. Current understanding is that hyperglycemia and other metabolic factors, such as hypertension and dyslipidemia, instigate a cascade of physiological and biochemical changes leading to retinal microvascular abnormalities, retinal ischemia, and resultant complications (**Figure 1**) (5). Upregulation of VEGF has been proven to be closely implicated in the pathogenesis of DR and its vascular complications such as neovascularization and DME (56). Subsequently, VEGF-independent pathways, such as erythropoietin, growth hormone and insulin-like growth factor, and angiopoietin, have been identified, through proteomic and other analyses (57, 58). Erythropoietin and its receptors are synthesized by retinal pigment epithelial cells and are important stimuli for mobilizing endothelial progenitor cells to impaired retinal sites (59, 60). Upregulation of erythropoietin expression in the ischemic retina may promote neovascularization and contribute to the progression of PDR (61). In one study, though the correlation between erythropoietin and VEGF levels were not strong, erythropoietin was more closely correlated with the presence of PDR than VEGF (57). Thus, erythropoietin inhibition has been proposed as a potential therapy option for DR, but the potential adverse effects on photoreceptor survival need to be balanced (57). Growth hormone and insulin-like growth factor may play a crucial role in pathological neovascularization in PDR and influence its progression (62). Growth hormone directly stimulates the proliferation of human retinal microvascular endothelial cells (63). Insulin-like growth factor and its binding protein are expressed in blood vessels, neurons, and glial cells throughout the retina and are altered in response to hyperglycemia and hypoxia (64). The angiopoietin pathway has already been trialed for therapeutic benefit. Faricimab, a novel bispecific antibody, provides dual inhibition of both VEGF-A, and angiopoietin-2 (Ang-2) to treat vascular eye diseases, including diabetic eye disease (65). It is thought that inhibition of Ang-2 works synergistically with VEGF inhibition, and helps to promote increased vascular stability (66). Recent phase III clinical trials seem to suggest that this approach may provide greater durability of treatment effect (67). Proteomic analyses have also shown raised levels of extracellular carbonic anhydrase in DR (68), which is thought to increase retinal vascular permeability, with equal potency to VEGF (58). Whether this pathway can be targeted for treatment with carbonic anhydrase inhibitors is an area for further study (69).

Furthermore, the traditional view that DR is purely a microvascular disease process is incomplete. The accumulating evidence indicates that there is a process of diabetic retinal neurodegeneration that accompanies or even precedes vascular damage. Evidence for loss of retinal neural elements can be seen as thinning of the retinal nerve fiber layer (RNFL) and ganglion cell layer on optical coherence tomography (OCT) imaging (70, 71). Functional abnormalities can also be demonstrated by electroretinography (ERG), including pattern ERG and multifocal ERG (72, 73). Some studies have also shown that these structural and functional neural abnormalities may develop early in DR, even before the onset of microvascular changes or retinopathy (74–76). In particular, the multifocal ERG has shown promise for detecting early abnormal alterations of retinal function in diabetic patients without apparent DR, and changes in multifocal ERG implicit time especially could be used as a potential clinical biomarker for providing early diagnosis of diabetic retinal disease and effective prognostication (75–77). It is postulated that chronic hyperglycemia induces retinal neurodegeneration, microvascular damage, and impairment of the neurovascular unit (61). The two key pathogenic factors involved in retinal neurodegeneration are the accumulation of extracellular glutamate, and imbalanced production of the retinal neuroprotective factors (78). The former is thought to result in neuron death (79), while the latter may impair the neuroprotective effect, which is related to the down regulation of neuroprotective factors such as pigment epithelium-derived factor, interstitial retinol-binding protein, somatostatin and several neurotrophins (78). Interestingly, there is some evidence that VEGF may be a survival factor for retinal neurons facing ischemic injury (80, 81). Anti-VEGF treatment certainly helps to reduce vascular leakage and retinal edema and improve visual acuity, but it has been postulated that there may be some deleterious effects on the retina from chronic long-term VEGF inhibition (82, 83). These potential limitations of anti-VEGF therapy need to be examined in further studies, and it would be interesting to see if multifocal ERG or other functional assessment modalities can be informative.

Finally, other pathogenic pathways are also likely to be important in DR, such as inflammation (84–86), increased oxidative stress (87–89), upregulation of receptors for advanced glycosylation end products (90–93), renin-angiotensin system (RAS) activation (88, 89, 94, 95) and dysfunctional endothelial progenitor cells (60). Much of the interaction between the neural and vascular abnormalities in the pathophysiology of DR remains to be clarified. However, as we better understand the relationship and link between these aspects, especially in the early stages of disease, such information will definitely influence our classification of DR, and may additionally promote the development of new potential treatment methods targeting these pathways (78, 96).

### Improved imaging technology and novel biomarkers

Major advancements have been made in retinal imaging technology over the past few decades. Up until the 1990s, the traditional retinal imaging modalities were standard color fundus photography (CFP), and fluorescein angiography, which were considered the gold standard for diagnosis, grading and visualization of retinal vasculature. Current DR fundus imaging patterns are summarized in **Table 2**. However, the development of better imaging techniques, such as OCT, ultra-widefield (UWF) imaging and optical coherence tomography angiography (OCTA), have allowed for new ways to visualize the anatomy of the retina and its vasculature, which will undoubtedly improve the ability to assess, prognosticate and monitor DR. **Table 3** summarizes the features of these new retinal imaging modalities in DR.

**Table 2:** Summary of current fundus imaging modalities in diabetic retinopathy.

Imaging modality	Advantages	Limitations	Clinical findings in DR
Fundus photography	Noncontact Wide application Gold standard for diagnosis and grading	Two-dimensional image Limited field of view Qualitative assessment	Microaneurysms Intraretinal haemorrhages Cotton-wool spot Venous beading Intraretinal microvascular abnormalities Neovascularization of optic disc (NVD) or elsewhere (NVE)
Fluorescein angiography (FA)	Gold standard for retinal vasculature Rapid assessment of retinal vascular changes High sensitivity when detecting low flow vascular lesions Differentiation of intraretinal microvascular anomalies (IRMAs) and neovascularization elsewhere (NVE) Differentiation of focal leak and diffuse capillary bed leak in DME Able to capture peripheral lesions Less liable to show artifacts than OCTA and easier to interpret	Invasive Two-dimensional image Time-consuming Potential adverse reactions to the dyes Leakage of dye can obscure details of vascular structures	Microaneurysms Retinal capillary non-perfusion Vascular telangiectasia Capillary drop outs Enlargement or irregularity of the foveal avascular zone The presence of neovascularization

**Table 3:** Summary of new multiple fundus imaging modalities in diabetic retinopathy.

Imaging modality	Advantages	Limitations	Clinical findings in DR
Optical coherence tomography (OCT)	Noncontact Widely used Cross-sectional and three-dimensional images Objective and quantitative assessment of DME Gold standard for diagnosis of DME and monitoring of treatment response	Fixation requirement Absence of visualizing vascular changes	Retinal thickness Subfoveal choroidal thickness Photoreceptor outer segment Hard exudates Hyperreflective retinal foci (HRF) Hyperreflective choroidal foci (HCF) Intraretinal cystoid spaces Disorganization of retinal inner layers (DRIL) Bridging retinal processes Subfoveal neurosensory detachment Integrity of ELM and EZ Taut posterior hyaloid membrane
Ultra-wide Field Retinal Imaging	Fast acquisition Noncontact High-resolution No pupillary dilatation Wide field of view Improvement of the detection of DR lesions Precise grading of DR	High cost and limited availability Image artifacts Peripheral distortion and magnification Superior and inferior periphery is not well visualized Difficulty to precisely measure the retinal surface area of lesions	Microaneurysms Intraretinal haemorrhages Cotton-wool spot Venous beading Intraretinal microvascular abnormalities Predominantly peripheral lesions Neovascularization of optic disc (NVD) or elsewhere (NVE) Preretinal haemorrhage Vitreous haemorrhage
Optical coherence tomography angiography (OCTA)	Quick and noncontact Cross-sectional and three-dimensional image Visualization and quantification of retinal vascular plexuses Visualization of vascular details Quantification of non-perfusion and vessel density Identification and monitoring of damage	High-resolution images need for good fixation Production of projection artifacts Limited peripheral view Complicate learning curve to capture and interpret images Not widely used	Microaneurysms Venous beading Decreased vascular density Capillary non-perfusion Enlargement of foveal avascular zone Increased vessel diameter index Decreased fractal dimension Increased vessel tortuosity Intraretinal microvascular anomalies (IRMAs) and neovascularization elsewhere (NVE)

### Optical coherence tomography

OCT is a non-contact and non-invasive imaging method that has become standard of care for diagnosis and monitoring of many retinal diseases (97). With the application of OCT for accurate retinal thickness measurements and imaging of retinal microstructure, new information about disease characteristics that were previously unrecognized is now available.

**Figure 2:**
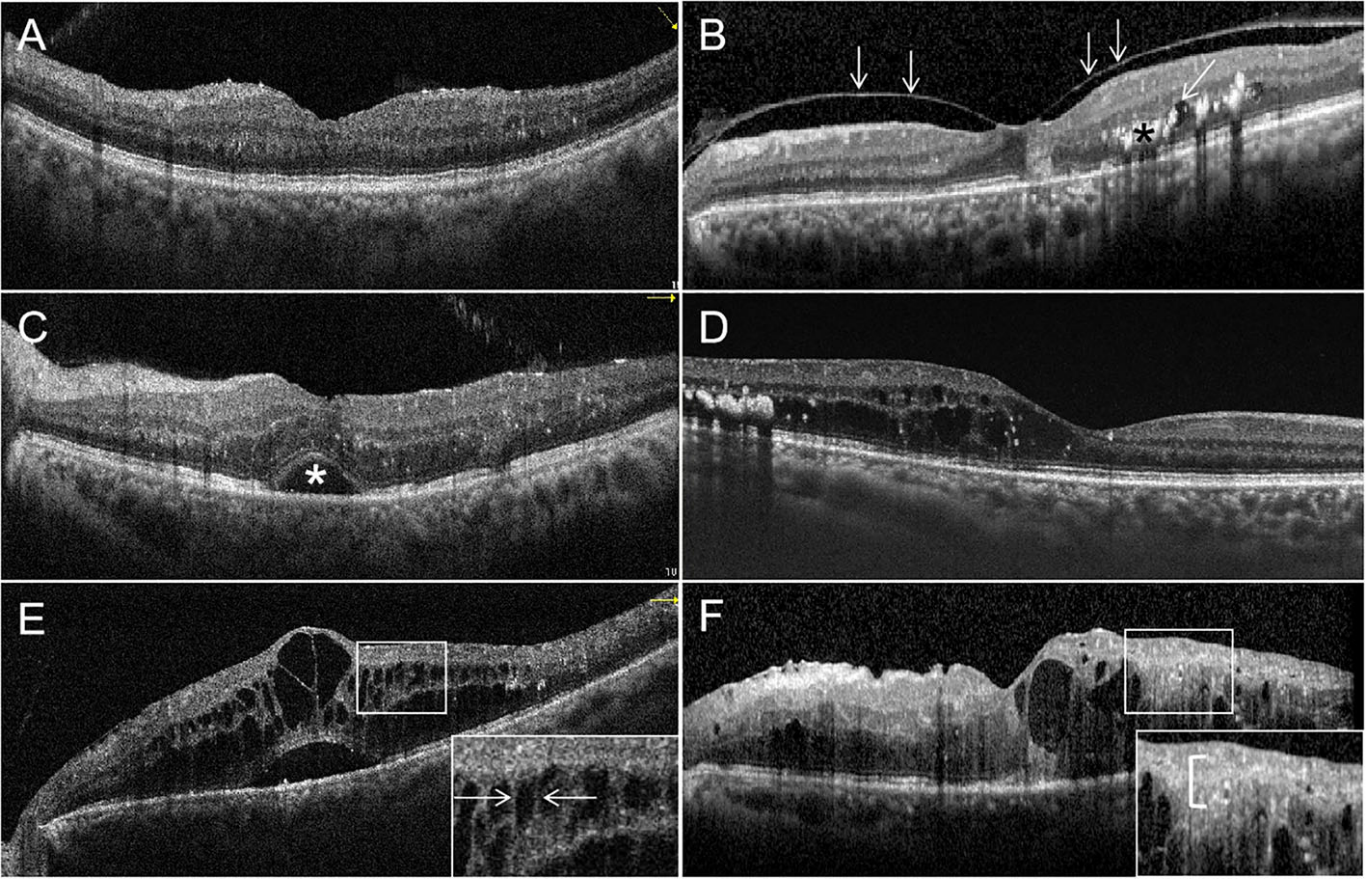
Optical coherence tomography (OCT) signs of diabetic macular edema (DME). **(A)** All retinal layers are intact and visible. The retinal profile is not altered. But there is diffuse macular thickening. **(B)** Vitreomacular traction with a thick posterior hyaloid membrane (white arrowheads), small cystoid spaces (oblique white arrowheads) and hard exudates (black asterisk) in the outer plexiform layer and the outer nuclear layer. **(C)** Multiple hyperreflective retinal foci (HRF) are seen. Subretinal fluid causing a neurosensory detachment of the fovea (white asterisk). **(D)** Cystic cavities, hard exudates, and HRF located in the outer retina, but the external limiting membrane (ELM) and ellipsoid zone (EZ) are intact. **(E)** The magnified image (white square) shows the bridging retinal processes (white arrowheads) between the cystic cavities. **(F)** Multiple cystoid spaces and HRF in the inner and outer layers with disorganization of the inner retinal layers (DRIL; white bracket in the magnified image). The ELM and EZ are disrupted under the fovea.

With OCT, a variety of potential biomarkers and structural abnormalities has been described in DR and DME. OCT can detect the significant reductions in the thickness of RNFL and ganglion cell-inner plexiform layer in DR, but also in diabetic patients without DR compared with healthy controls (98). This retinal thinning is thought to represent diabetic retinal neurodegeneration or neural dysfunction (51). Other quantitative changes that have been described include reduced retinal thickness, retinal volume, and decreased optical reflectivity (99). Qualitative abnormalities are also detectable, such as the presence of intraretinal hyper-reflective foci (HRF) that are thought to represent microglial activation and migration. More HRF have been shown to be present in diabetic patients with DR compared to those without DR, and has been associated with the progression of DR (100). Some OCT biomarkers have been associated with visual acuity outcomes in DME and DR, such as disruption of the external limiting membrane (ELM) (101) and ellipsoid zone (EZ) (102), and disorganization of retinal inner layers (DRIL). DRIL has also been shown to be associated with increased severity of DR (103). In addition, other potential biomarkers in characterizing DR include hyperreflective choroidal foci (HCF), intraretinal cystoid spaces, hard exudates, and subfoveal neurosensory detachment, which are shown in **Figure 2**. However, prospective validation is needed before many of these potential biomarkers can be useful tools in clinical practice.

Based on OCT changes and biomarkers in DR and DME, some groups have proposed OCT-based classification systems for DME or diabetic maculopathy. One such classification describes different types of DME including the sponge-like retinal swelling type, cystoid macular edema type, and serous retinal detachment type (104, 105). Another, more comprehensive, classification takes into account multiple different OCT biomarkers, to classify diabetic maculopathy into four different stages: early diabetic maculopathy (DM), advanced DM, severe DM, and atrophic maculopathy (106).

### Ultra-widefield retinal imaging

**Figure 3:**
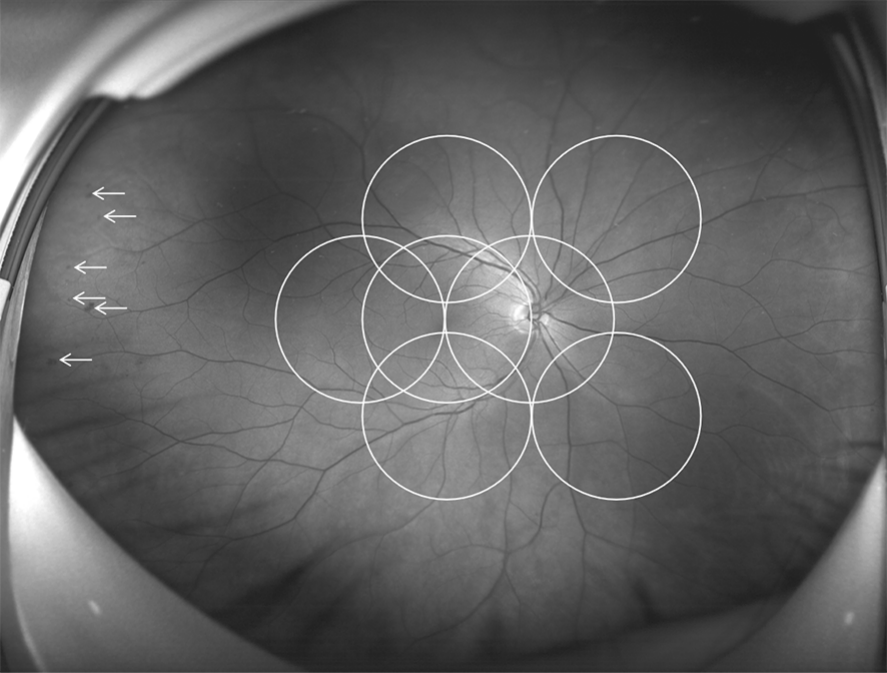
Comparison of an ultra-wide field (UWF) retinal image and the Early Treatment Diabetic Retinopathy Study (ETDRS) 7 standard photographic fields. UWF retinal image is superimposed by the ETDRS 7 standard fields in white circles. The white arrowheads showing diabetic retinopathy lesions predominantly peripheral to the ETDRS fields.

**Figure 4:**
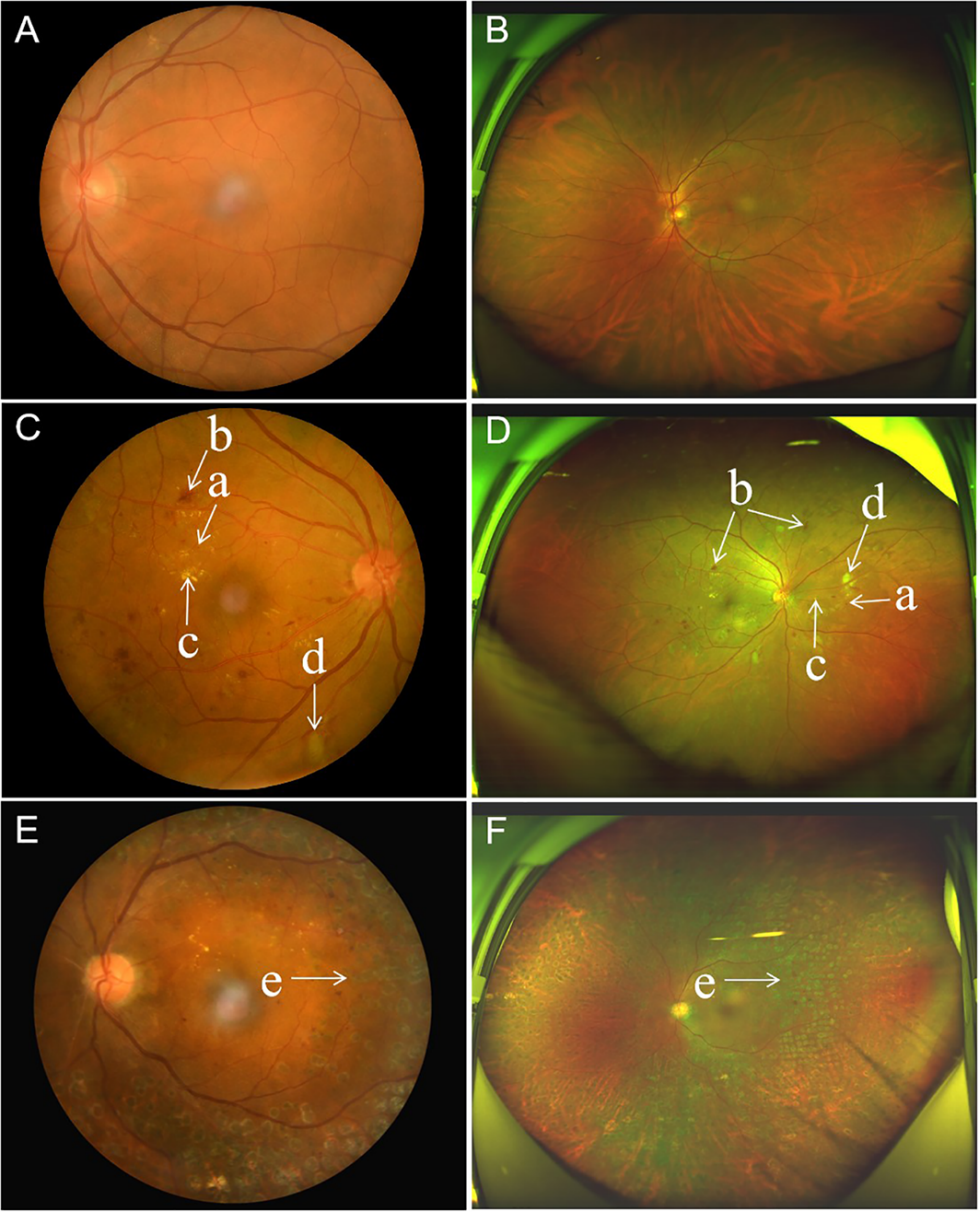
Comparison of paired standard 45° fundus photographs and ultra-widefield photographs in three diabetic patients. **(A, B)**, Standard 45° fundus photograph and ultra-widefield photograph from the left eye of the same patient, with no diabetic retinopathy. **(C, D)**, Standard 45° fundus photograph showing microaneurysms, hard exudate, cotton wool spots and dot-blot retinal hemorrhages from diabetic retinopathy in the posterior pole, and accompanying ultra-widefield photograph from the same eye showing more retinal lesions in the periphery. **(E, F)**, Standard 45° fundus photograph showing an eye with diabetic retinopathy that has undergone panretinal laser photocoagulation, and the accompanying ultra-widefield photograph from the same eye showing the peripheral extent of the laser photocoagulation scars. **(a)** microaneurysms, **(b)** hemorrhage, **(c)** hard exudate, **(d)** cotton wool spots, and **(e)** photocoagulation scars.

**Figure 5:**
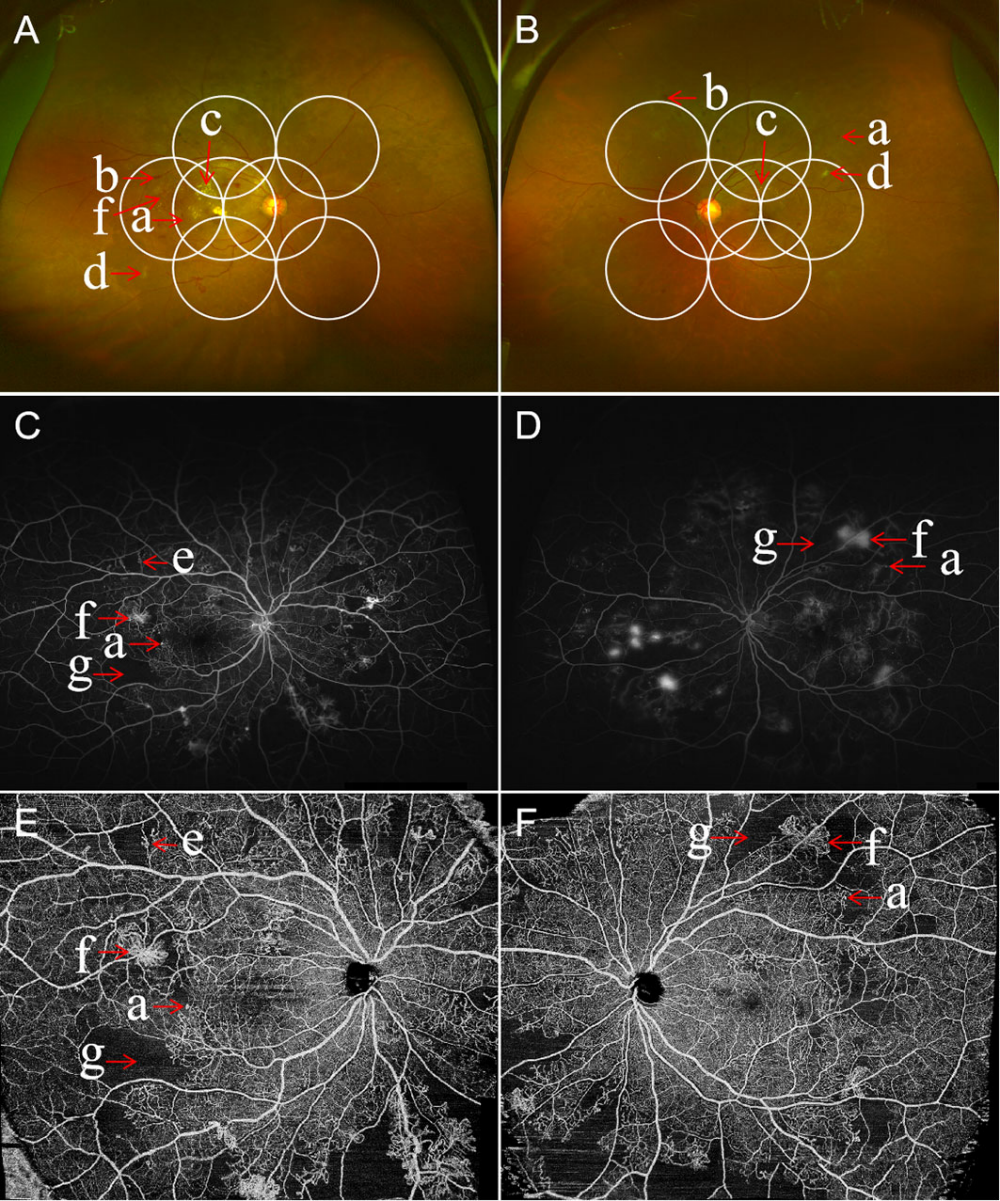
Multimodal images of proliferative diabetic retinopathy in both eyes of the same patient. Ultra-wide field (UWF) retinal images with the ETDRS 7-feld, 30-degree fundus images in circles outlined in white. The UWF fundus imaging of right eye **(A)** and left eye **(B)** showing retinal hemorrhages, microaneurysms, hard exudates, cotton wool spots, abnormal vascular loop, intraretinal microvascular abnormalities (IRMA), and retinal neovascularization. The ultra-widefield fluorescein angiography of right eye **(C)** and left eye **(D)** illustrating the corresponding hyperfluorescent dots of microaneurysms, areas of capillary non-perfusion, and multiple small areas of neovascularization identified by the hyperfluorescent leakage of dye. Corresponding wide field swept-source optical coherence tomography angiography (WF SS-OCTA) of right eye **(E)** and left eye **(F)** exhibiting area of non-perfusion, abnormal vascular loop, IRMA, and retinal neovascularization. **(a)** microaneurysms, **(b)** hemorrhage, **(c)** hard exudates, **(d)** cotton wool spots, **(e)** IRMA, **(f)** retinal neovascularization, and **(g)** areas of retinal ischemia.

UWF imaging is defined as retinal imaging providing at least 110°field of view, with visualization including the anterior edge of the vortex vein ampullae (107), though many current commercials systems can capture up to 200° in a single retinal image. **Figure 3** shows an UWF retinal image in comparison with the area covered by 7 standard-field CFP images. Although the ETDRS classification has been the gold standard for DR classification and detection for many years, it is important to remember that a single 45° CFP image only covers about 15% of retinal surface area, and the 7 standard fields in total cover about 30% (108). In contrast, UWF images can cover about 82% of total retinal surface area (**Figure 4**). Recent studies examining UWF imaging in DR have shown that more than 50% of DR graded lesions are located outside the area covered by the 7 standard ETDRS fields, and they also demonstrate that peripheral DR lesions may have powerful prognostic significance (49). One study showed that eyes with predominantly peripheral lesions (PPLs) had a 3.2-fold increased risk of DR progression and a 4.7-fold increased risk of PDR progression compared to eyes without PPLs (109). Meanwhile, UWF imaging has shown that the PPLs outside the ETDRS fields account for 40% in the eyes with DR and that PPLs may lead to a more severe level ETDRS grading in about 10% eyes (**Figures 5A, B**) (110). In one study, about 50% of neovascularization (new vessels elsewhere) was predominantly peripheral when examined with UWF images (111). Clearly, the peripheral retina as visualized by UWF imaging can provide valuable information about the classification and progression of DR, and visual prognosis, but how to this should be incorporated into a new DR classification is currently unclear.

UWF imaging can also be applied to fluorescein angiography. UWF fluorescein angiography (UWFA), together with color or pseudocolor UWF imaging, has been applied to detect peripheral neovascularization and ischemic areas, and to guide the diagnosis and treatment of DR (**Figures 5C, D**). In one study on UWFA, parameters such as the areas of non-perfusion, neovascularization and panretinal photocoagulation scars displayed by UWFA images increased by 3.9 times, 1.9 times and 3.8 times, respectively compared with 7 standard field ETDRS images.

Meanwhile, Ehlers et al. demonstrated the relationship between the quantitative angiographic parameters of microaneurysm count, panretinal leakage, and ischemic area on UWFA, and the clinical severity of DR (112). Such parameters derived from UWF photos and UWFA may be used as biomarkers to assess the objective information that may be related to need for therapeutic intervention or therapeutic response.

### Optical coherence tomography angiography

**Figure 6:**
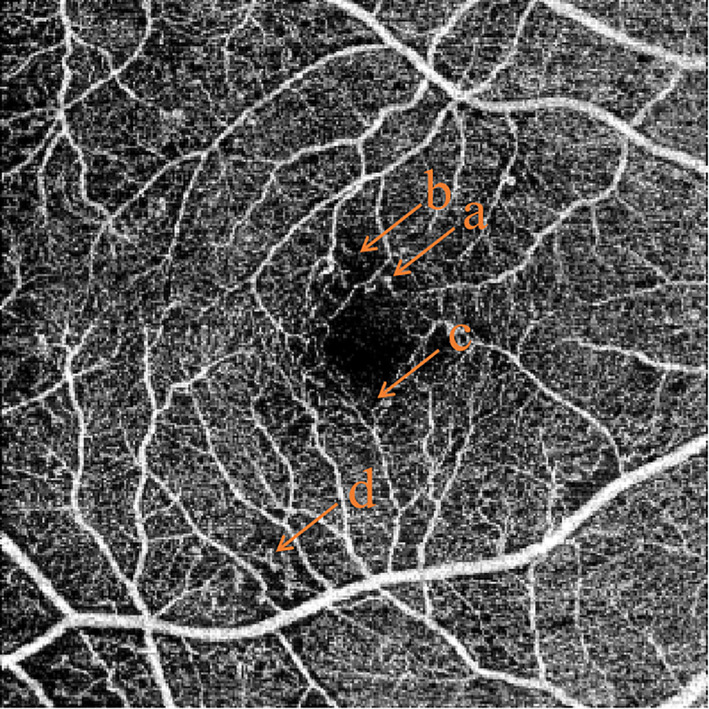
Common features of OCTA in non-proliferative diabetic retinopathy. **(A)** microaneurysms, **(B)** capillary non-perfusion area, **(C)** slightly enlarged foveal avascular zone, **(D)** abnormal vascular loops.

OCTA is a novel, non-contact and non-invasive technique capable of capturing high-resolution images of the retinal and choroidal vessels (113, 114). OCTA displays vascular flow information by creating three-dimensional depth-resolved images of the retinal and choroidal vascular system, so as to identify areas with or without flow, which is an important aspect of DR assessment. Although OCTA cannot reveal vascular leakage, it still has many advantages over fluorescein angiography (FA) (115). Most importantly, OCTA is non-invasive, and can provide detailed information about the retinal microvasculature in DR, without the need for intravenous contrast dye (**Figure 6**) (116, 117). Meanwhile, the acquisition of OCTA image and data is more convenient and rapid than FA. Furthermore, OCTA provides depth-resolved images, and can allow separate visualization of the superficial, middle and deep retinal capillary plexuses, which may provide additional pathological information over traditional dye-based angiography (115).

**Figure 7:**
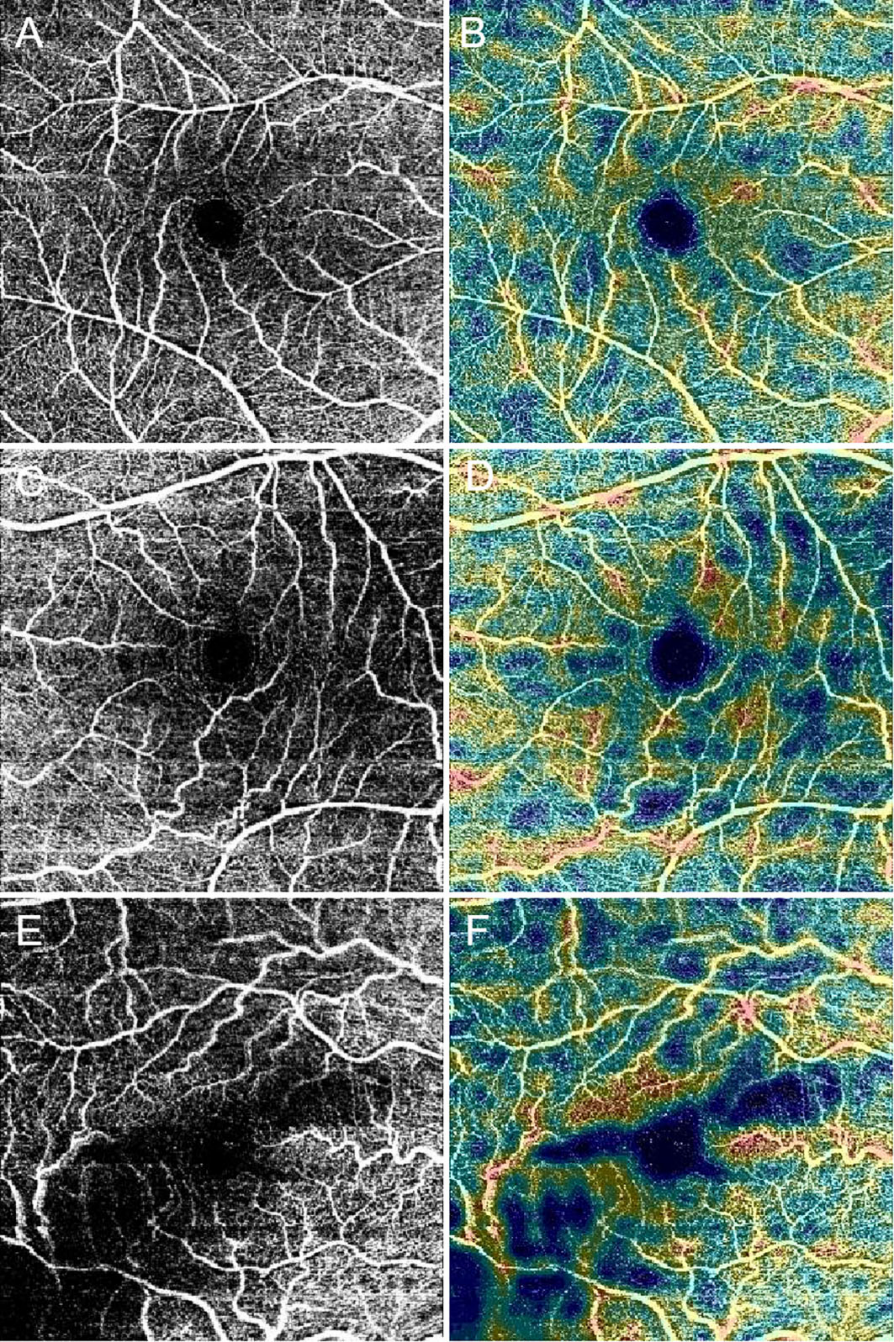
Optical coherence tomography angiography (OCTA) images present the foveal avascular zone, macular capillary nonperfusion and vessel density in diabetic patients. Black and white scans **(A**, **C**, and **E)** represent OCTA angiograms. Color map scans **(B**, **D**, and **F)** represent color-coded vessel density in the corresponding OCTA angiograms. With worsening diabetic retinopathy severity level, the foveal avascular zone diameters increase, and the non-perfusion area and the vessel density decrease in these images. **(A, B)**, No diabetic retinopathy. **(C, D)**, nonproliferative diabetic retinopathy (NPDR). **(E, F)**, Proliferative diabetic retinopathy (PDR).

Vascular changes associated with diabetes can be detected by OCTA even before the appearance of clinically-visible DR (118). Some of the parameters provided by OCTA include vessel density, vessel tortuosity and fractal dimension, of the superficial capillary plexus, deep capillary plexus, and the middle capillary plexus (119). OCTA can also identify foveal avascular zone parameters such as size, circularity and perimeter (**Figure 7**). Many such parameters have been correlated with severity of DR (120). Although FA has a higher sensitivity than OCTA in detecting microaneurysms, some studies have proven that OCTA can detect microaneurysms that are not detectable by FA (121, 122). Meanwhile, OCTA can also detect intraretinal microvascular anomalies (IRMAs), neovascularization of the disc (NVD), and neovascularization elsewhere (NVE) in intraretinal and extraretinal neovascularization with excellent reliability (**Figure 5E, F**) (121, 123). Not only does OCTA provide better detection of IRMAs and neovascularizations compared to FA and CFP, but it also allows for better morphologic characterization of IRMA and NV, because of the absence of late dye leakage. Meanwhile, both widefield OCTA and UWFA have been compared and applied in patients with DR. One study suggested that widefield OCTA had a higher detection rate of capillary non-perfusion areas than ultrawide field fluorescein angiography (124). One research group has proposed a new staging system for DR based on wide-field swept-source OCTA. This classification uses various retinal vascular and structural features to define various disease stages including no DR, subclinical DR, non-proliferative DR, pre-proliferative DR, PDR, and tractional retinal detachment (125). Naturally, such classification systems will need to be validated and refined over time, and new technological advances in OCTA technology will also influence these modifications.

### Artificial intelligence and deep learning

Artificial intelligence (AI) was originally proposed in 1956, as a field of study looking to develop computer methods to simulate human intelligence and perform complex cognitive tasks. Deep learning (DL) is a subset of AI, which is designed to mimic neural networks in the human brain, enabling systems to cluster and learn from unstructured data, using this make classification decisions and predictions with incredible accuracy. Today, DL has been widely used in various medical and clinical settings. Particularly within ophthalmology, AI using DL has been adopted by a variety of groups to develop algorithms for automated DR diagnosis and screening. **Supplementary Table 1** provides a summary of the AI systems in detection of DR using fundus photographs, UWF fundus images, and OCTA images.

The first DL algorithms for automated DR detection were developed by Gulshan et al. in 2016 (126), and Ting et al. in 2017 (127). These algorithms used standard CFP images as input. Both groups demonstrated that the algorithms had high diagnostic accuracy, with areas under the receiving operating characteristic curves of more than 0.9 on independent datasets. Since then, numerous DL algorithms have been developed for this purpose, and there are multiple that have already received regulatory approval, and are in clinical use. For example, IDx-DR (IDx LLC, Coralville, IA, USA) and EyeArt (Eyenuk, Inc., Woodland Hills, CA. USA) have received USA Food and Drug Administration approval (128, 129), while SELENA+ (EyRIS, Singapore) has received European CE Mark approval. In terms of DL for other imaging modalities, Cheung et al. recently developed an effective DL algorithm for DR detection on UWF images, using a dataset of 9,392 images from 4 different countries (130). As for OCTA, Ryu et al. evaluated the role of DL in diagnosing DR in OCTA images (131). Their DL model could achieve an overall accuracy, sensitivity, and specificity of 91-98%, 86-97%, 94-99%. Automated analysis of different imaging modalities with AI and DL is now possible, and validation and implementation of these algorithms is likely to greatly improve and optimize the efficiency and of DR screening and diagnosis.

AI and DL could feature in a new DR classification system in a few ways. First, if AI-based automated grading is equivalent or better than human graders in terms of accuracy and reproducibility, then a new DR classification system could accept AI-based grading for use in research and clinical practice. Second, AI could be used to optimize or improve prognostication of patient outcomes, over and above existing risk stratification methods. This may be through image analysis, or through the addition or inclusion of multimodal clinical data as well. Third, if classification systems become more quantitative, AI could be used to automate the lesion quantification and counting processes. Nevertheless, significant barriers still remain in this area. Developing and validating robust AI algorithms requires good longitudinal datasets. As new imaging modalities are developed or included, we would need new large datasets of these images, linked to outcomes of interest, in order to develop these AI models. Explainability and clinician acceptance of AI models in clinical practice is also an area that can be improved.

### Quantitative assessment of diabetic retinopathy

Current DR classification systems are semi-quantitative and categorical. For example, in the ETDRS, DR lesions such as H/Mas or IRMAs are graded individually based on their severity, which is based on comparison against reference standard photographs. The more lesions such as H/Mas that an eye has, the greater the severity, but the severity is divided into a few severity categories, and is not a continuous quantitative scale. The classification systems were designed this way, because it was not practical at the time to individually count lesions for classification. However, it is possible that objective quantification of lesions and other biomarkers, such as OCTA vessel density or UWFA ischemic areas may provide more accurate disease evaluation and better prediction of treatment response.

For example, Sadda et al. demonstrated that quantitative assessment of DR lesions on UWF images identified new risk factors for DR progression, such as hemorrhage surface area or distance of hemorrhages from the optic nerve head (132). Sears et al. compared subjective and quantitative methods of determining PPLs and the distribution of DR lesion in UWF images, and found that objective quantitative assessment of DR was more accurate. On UWFA (133), Sun et al. analyzed quantitative parameters related to leakage, ischemia and microaneurysm counts, and found that they were strongly associated with DR severity (134), as well as PDR and DME (135). On OCTA, Alam et al. characterized quantitative OCTA features of NPDR and observed that quantitative OCTA metrics such as blood vessel density could be effective for quantification and staging of NPDR (136).

With AI, automated quantification of relevant parameters and metrics from retinal photographs and other imaging modalities is now possible. Quantitative assessment and staging may provide more accurate prognostication for DR outcomes, but this will need to be validated and evaluated in future studies. Also, there are multiple different imaging techniques that can be analyzed quantitatively in DR, and standardization of quantitative method is likely to be important going forward.

### Response to new treatments

Our DR classifications at present are all based on grading the presence and severity of visible retinal lesions and photographic appearance. Up until the last decade, the mainstay of treatment for DR was PRP, to reduce the risk of progression to PDR, and therefore to reduce the risk of severe visual loss. After successful PRP, characteristic DR lesions such as H/Mas and neovascularization tend to regress, and our existing DR classification systems cannot be formally applied to prognosticate such eyes that have undergone disease-modifying treatment. There was no strong need to develop a formal classification for such post-PRP eyes, as the effect of PRP in reducing retinal ischemia was persevering and long-lasting. However, this is no longer true with new treatments that we have for DR and DME now. Treatments such as intravitreal anti-VEGF and corticosteroid injections, are known to modify the appearance of the fundus in patients with DR (137–140). Many patients show “improvement” in DR severity scales if these DR lesions regress. However, none of these therapies effectively address the underlying problem of retinal ischemia (124). Thus, the disease tends to recur or progresses rapidly after stopping treatment. Current classification systems may not be applied to accurately prognosticate these post-treatment eyes, and so this is a major need to be addressed in a new classification.

Current classification systems are also based primarily on progression to PDR, which used to be the major cause of visual loss in DR. However, DME is now the leading cause of visual impairment in DR, and there are effective treatments for DME (141). Furthermore, up to 40 to 50% of eyes with DME do not respond fully to anti-VEGF treatment (142), and it has been suggested that different DME phenotypes determined by OCT appear to have different prognosis and responsiveness to treatment (143). Therefore, an effective updated classification system should also include risk stratification and severity gradings for DME.

## Conclusion

Diabetic retinopathy is a complex, multifactorial disease, and our understanding of this disease is constantly evolving. Over the years, our DR classification systems have gone through various iterations, and have had to be modified and updated to keep up with our understanding of the disease, and with technological advancements. Though our current ETDRS and ICDR severity scales have provided the foundation for major research trials and modern clinical management of DR, it is time for an update. The significant advances that have been made over the past few decades in disease pathophysiology, imaging technology, artificial intelligence and treatment, must inform a new classification system. New DR classification systems should be based on available evidence and robustly validated, and will hopefully translate to better outcomes and managements for the millions of patients with DR worldwide.

## Author contributions

All authors contributed to the article and approved the submitted version.
