# Supplementary material for: Classification of diabetic retinopathy: Past, present and future
Source: Front Endocrinol (Lausanne). 2022 Dec 16;13:1079217. doi: 10.3389/fendo.2022.1079217 (PMC9800497; doi:10.3389/fendo.2022.1079217)
Supplement: Supplementary file 1 [file Table_1.docx]

Supplementary Material

# Supplementary Table 1. Artificial intelligence systems for automated detection of diabetic retinopathy.

| Author | Year | AI system | Algorithm | Imaging modality | Race | Dataset for development | AUC | Sensitivity | Specificity |  |
| --- | --- | --- | --- | --- | --- | --- | --- | --- | --- | --- |
| Lee et al. (144) | 2021 | OphtAI AirDoc Eyenuk Retina-AI Health Retmarker | Image analysis technology | Fundus photo | White African American Asian Other | 311,604 | N/A | 80.47% | 81.28% |  |
| Wang et al. (145) | 2021 | N/A | Inception-v3 | Fundus photo | Chinese | 10,678 | 0.943-0.938 | 90.5%-90.6% | 78.5%-80.7% |  |
| Vaghefi et al. (146) | 2021 | THEIA | N/A | Fundus photo | White | 160,585 | N/A | 94% | 63% |  |
| Oh et al. (147) | 2021 | N/A | ResNet-34 | UWF fundus images | Asian | 11,734 | 0.915 | 83.38% | 83.41% |  |
| Lu et al. (148) | 2021 | N/A | CNN | Fundus photo | Chinese | 41,866 | 0.9824 | 90.03% | 96.09% |  |
| Ipp et al. (149) | 2021 | EyeArt | N/A | Fundus photo | White Asian Other | 1054 | N/A | 95.50% | 85.00% |  |
| Dai et al. (150) | 2021 | DeepDR system | ResNet Mask-RCNN | Fundus photo | Chinese | 466,247 | 0.973 | 94.10% | 89.70% |  |
| Wang et al. (151) | 2020 | N/A | CNN | OCTA images | Chinese | 28,664 | N/A | 96.39% | 98.91% |  |
| Alyoubi et al. (152) | 2021 | N/A | CNN | Fundus photo | Aegypti | 13,673 | N/A | 89% | 97.30% |  |
| Zhang et al. (153) | 2020 | VoxelCloud Retina | ResNet v2 | Fundus photo | Chinese | 94,199 | N/A | 83.30% | 92.50% |  |
| Shaban et al. (154) | 2020 | N/A | CNN | Fundus photo | White | 3,648 | N/A | 87%-89% | 94%-95% |  |
| Heydon et al. (129) | 2021 | EyeArt | N/A | Fundus photo | White Asian Black | 30,405 | N/A | 95.70% | 68% |  |
| Limwattanayingyong et al. (155) | 2020 | N/A | Image analysis technology | Fundus photo | White Asian | 5,738 | N/A | 95.03% | 97.97% |  |
| Heisler et al. (156) | 2020 | N/A | VGG19 ResNet50 DenseNet | OCTA images | White | 463 | N/A | 90.41% | 93.33% |  |
| Burlina et al. (157) | 2020 | EyePACS | ResNet50 | Fundus photo | White | 88,692 | 0.8330 | N/A | N/A |  |
| Stevenson et al. (158) | 2019 | MedicMind | Inception-v3 | Fundus photo | White | 4,435 | 0.58 | 75% | 89% |  |
| Liu et al. (159) | 2019 | N/A | CNN | Fundus photo | Chinese | 60,000 | 0.9823 | 90.94% | 95.74% |  |
| Ruamviboonsuk et al. (160) | 2019 | N/A | Inception-v4 | Fundus photo | Thai | 25,326 | 0.987 | 96.80% | 95.60% |  |
| He et al. (161) | 2020 | Airdoc | Inception-v4 | Fundus photo | Chinese | 3,556 | 0.95 | 91.80% | 98.79% |  |
| Gulshan et al. (162) | 2019 | EyePACS | N/A | Fundus photo | Indian | 3,779 | 0.98 | 92.10% | 95.20% |  |
| Bhaskaranand et al. (163) | 2019 | EyeArt | Image analysis technology | Fundus photo | White | 850,908 | 0.965 | 91.30% | 91.10% |  |
| Li et al. (164) | 2018 | EyePACS | N/A | Fundus photo | Chinese | 106,244 | 0.989 | 97.00% | 91.40% |  |
| Sayres et al. (165) | 2019 | EyePACS | Inception-v4 | Fundus photo | White | 1,796 | N/A | 91.50% | 94.70% |  |
| Keel et al. (166) | 2018 | EyeGrader | inception-v3 | Fundus photo | White | 66,790 | N/A | 92.30% | 93.70% |  |
| Ting et al. (127) | 2017 | N/A | CNN | Fundus photo | Chinese Indian Malay | 494,661 | 0.936 | 90.50% | 91.60% |  |
| Gargeya et al. (167) | 2017 | EyePACS | CNN | Fundus photo | White | 75,137 | 0.97 | 94% | 98% |  |
| Gulshan et al. (168) | 2016 | Google | inception-v3 | Fundus photo | White | 128,175 | 0.990-0.991 | 87.00%-97.50% | 93.9%-98.5% |  |
| Abràmoff et al. (169) | 2016 | IDx-DR X2.1 | CNN | Fundus photo | White | 1,748 | 0.98 | 96.80% | 87.00% |  |
| Hansen et al. (170) | 2015 | IDP | Non-DL | Fundus photo | White | 6,788 | 0.878 | 86.70% | 70.00% |  |
| Abràmoff et al. (171) | 2013 | IDP | Non-DL | Fundus photo | White | 1,748 | 0.937 | 96.80% | 59.40% |  |
| AUC Area under the curve, N/A Not available, UWF Ultra-wide field, CNN Convolutional neural network, OCTA Optical coherence tomography angiography, IDP Iowa Detection Program, DL Deep learning | | | | | | | | | |  |
|  |  |  |  |  |  |  |  |  |  |  |

**References:**

127. Ting DSW, Cheung CY, Lim G, Tan GSW, Quang ND, Gan A, et al. Development and Validation of a Deep Learning System for Diabetic Retinopathy and Related Eye Diseases Using Retinal Images from Multiethnic Populations with Diabetes. *JAMA* (2017) 318(22):2211-23. doi: 10.1001/jama.2017.18152.

129. Heydon P, Egan C, Bolter L, Chambers R, Anderson J, Aldington S, et al. Prospective Evaluation of an Artificial Intelligence-Enabled Algorithm for Automated Diabetic Retinopathy Screening of 30 000 Patients. *Br J Ophthalmol* (2021) 105(5):723-8. doi: 10.1136/bjophthalmol-2020-316594.

144. Lee A, Yanagihara R, Lee C, Blazes M, Jung H, Chee Y, et al. Multicenter, Head-to-Head, Real-World Validation Study of Seven Automated Artificial Intelligence Diabetic Retinopathy Screening Systems. *Diabetes Care* (2021) 44(5):1168-75. doi: 10.2337/dc20-1877.

145. Wang Y, Yu M, Hu B, Jin X, Li Y, Zhang X, et al. Deep Learning-Based Detection and Stage Grading for Optimising Diagnosis of Diabetic Retinopathy. *Diabetes Metab Res Rev* (2021) 37(4):e3445. doi: 10.1002/dmrr.3445.

146. Vaghefi E, Yang S, Xie L, Hill S, Schmiedel O, Murphy R, et al. Theia™ Development, and Testing of Artificial Intelligence-Based Primary Triage of Diabetic Retinopathy Screening Images in New Zealand. *Diabet Med* (2021) 38(4):e14386. doi: 10.1111/dme.14386.

147. Oh K, Kang HM, Leem D, Lee H, Seo KY, Yoon S. Early Detection of Diabetic Retinopathy Based on Deep Learning and Ultra-Wide-Field Fundus Images. *Sci Rep* (2021) 11(1):1897. doi: 10.1038/s41598-021-81539-3.

148. Lu L, Ren P, Lu Q, Zhou E, Yu W, Huang J, et al. Analyzing Fundus Images to Detect Diabetic Retinopathy (Dr) Using Deep Learning System in the Yangtze River Delta Region of China. *Ann Transl Med* (2021) 9(3):226. doi: 10.21037/atm-20-3275.

149. Ipp E, Liljenquist D, Bode B, Shah V, Silverstein S, Regillo C, et al. Pivotal Evaluation of an Artificial Intelligence System for Autonomous Detection of Referrable and Vision-Threatening Diabetic Retinopathy. *JAMA Netw Open* (2021) 4(11):e2134254. doi: 10.1001/jamanetworkopen.2021.34254.

150. Dai L, Wu L, Li H, Cai C, Wu Q, Kong H, et al. A Deep Learning System for Detecting Diabetic Retinopathy across the Disease Spectrum. *Nat Commun* (2021) 12(1):3242. doi: 10.1038/s41467-021-23458-5.

151. Wang L, Wang G, Zhang M, Fan D, Liu X, Guo Y, et al. An Intelligent Optical Coherence Tomography-Based System for Pathological Retinal Cases Identification and Urgent Referrals. *Transl Vis Sci Technol* (2020) 9(2):46. doi: 10.1167/tvst.9.2.46.

152. Alyoubi W, Abulkhair M, Shalash WJS. Diabetic Retinopathy Fundus Image Classification and Lesions Localization System Using Deep Learning. *Sensors (Basel)* (2021) 21(11). doi: 10.3390/s21113704.

153. Zhang Y, Shi J, Peng Y, Zhao Z, Zheng Q, Wang Z, et al. Artificial Intelligence-Enabled Screening for Diabetic Retinopathy: A Real-World, Multicenter and Prospective Study. *BMJ Open Diabetes Res Care* (2020) 8(1). doi: 10.1136/bmjdrc-2020-001596.

154. Shaban M, Ogur Z, Mahmoud A, Switala A, Shalaby A, Abu Khalifeh H, et al. A Convolutional Neural Network for the Screening and Staging of Diabetic Retinopathy. *PloS One* (2020) 15(6):e0233514. doi: 10.1371/journal.pone.0233514.

155. Limwattanayingyong J, Nganthavee V, Seresirikachorn K, Singalavanija T, Soonthornworasiri N, Ruamviboonsuk V, et al. Longitudinal Screening for Diabetic Retinopathy in a Nationwide Screening Program: Comparing Deep Learning and Human Graders. *J Diabetes Res* (2020) 2020:8839376. doi: 10.1155/2020/8839376.

156. Heisler M, Karst S, Lo J, Mammo Z, Yu T, Warner S, et al. Ensemble Deep Learning for Diabetic Retinopathy Detection Using Optical Coherence Tomography Angiography. *Transl Vis Sci Technol* (2020) 9(2):20. doi: 10.1167/tvst.9.2.20.

157. Burlina P, Paul W, Mathew P, Joshi N, Pacheco K, Bressler NJJo. Low-Shot Deep Learning of Diabetic Retinopathy with Potential Applications to Address Artificial Intelligence Bias in Retinal Diagnostics and Rare Ophthalmic Diseases. *JAMA Ophthalmol* (2020) 138(10):1070-7. doi: 10.1001/jamaophthalmol.2020.3269.

158. Stevenson C, Hong S, Ogbuehi KJC, ophthalmology e. Development of an Artificial Intelligence System to Classify Pathology and Clinical Features on Retinal Fundus Images. *Clin Exp Ophthalmol* (2019) 47(4):484-9. doi: 10.1111/ceo.13433.

159. Liu Y, Li Z, Xu C, Li J, Liang RJAiim. Referable Diabetic Retinopathy Identification from Eye Fundus Images with Weighted Path for Convolutional Neural Network. *Artif Intell Med* (2019) 99:101694. doi: 10.1016/j.artmed.2019.07.002.

160. Raumviboonsuk P, Krause J, Chotcomwongse P, Sayres R, Raman R, Widner K, et al. Deep Learning Versus Human Graders for Classifying Diabetic Retinopathy Severity in a Nationwide Screening Program. *NPJ Digit Med* (2019) 2:25. doi: 10.1038/s41746-019-0099-8.

161. He J, Cao T, Xu F, Wang S, Tao H, Wu T, et al. Artificial Intelligence-Based Screening for Diabetic Retinopathy at Community Hospital. *Eye (Lond)* (2020) 34(3):572-6. doi: 10.1038/s41433-019-0562-4.

162. Gulshan V, Rajan R, Widner K, Wu D, Wubbels P, Rhodes T, et al. Performance of a Deep-Learning Algorithm Vs Manual Grading for Detecting Diabetic Retinopathy in India. *JAMA Ophthalmol* (2019) 137(9):987-93. doi: 10.1001/jamaophthalmol.2019.2004.

163. Bhaskaranand M, Ramachandra C, Bhat S, Cuadros J, Nittala MG, Sadda SR, et al. The Value of Automated Diabetic Retinopathy Screening with the Eyeart System: A Study of More Than 100,000 Consecutive Encounters from People with Diabetes. *Diabetes Technol Ther* (2019) 21(11):635-43. doi: 10.1089/dia.2019.0164.

164. Li Z, Keel S, Liu C, He Y, Meng W, Scheetz J, et al. An Automated Grading System for Detection of Vision-Threatening Referable Diabetic Retinopathy on the Basis of Color Fundus Photographs. *Diabetes care* (2018) 41(12):2509-16. doi: 10.2337/dc18-0147.

165. Sayres R, Taly A, Rahimy E, Blumer K, Coz D, Hammel N, et al. Using a Deep Learning Algorithm and Integrated Gradients Explanation to Assist Grading for Diabetic Retinopathy. *Ophthalmology* (2019) 126(4):552-64. doi: 10.1016/j.ophtha.2018.11.016.

166. Keel S, Lee P, Scheetz J, Li Z, Kotowicz M, MacIsaac R, et al. Feasibility and Patient Acceptability of a Novel Artificial Intelligence-Based Screening Model for Diabetic Retinopathy at Endocrinology Outpatient Services: A Pilot Study. *Sci Rep* (2018) 8(1):4330. doi: 10.1038/s41598-018-22612-2.

167. Gargeya R, Leng TJO. Automated Identification of Diabetic Retinopathy Using Deep Learning. *Ophthalmology* (2017) 124(7):962-9. doi: 10.1016/j.ophtha.2017.02.008.

168. Gulshan V, Peng L, Coram M, Stumpe MC, Wu D, Narayanaswamy A, et al. Development and Validation of a Deep Learning Algorithm for Detection of Diabetic Retinopathy in Retinal Fundus Photographs. *JAMA* (2016) 316(22):2402-10. doi: 10.1001/jama.2016.17216.

169. Abràmoff M, Lou Y, Erginay A, Clarida W, Amelon R, Folk J, et al. Improved Automated Detection of Diabetic Retinopathy on a Publicly Available Dataset through Integration of Deep Learning. *Invest Ophthalmol Vis Sci* (2016) 57(13):5200-6. doi: 10.1167/iovs.16-19964.

170. Hansen M, Abràmoff M, Folk J, Mathenge W, Bastawrous A, Peto TJPo. Results of Automated Retinal Image Analysis for Detection of Diabetic Retinopathy from the Nakuru Study, Kenya. *PLoS One* (2015) 10(10):e0139148. doi: 10.1371/journal.pone.0139148.

171. Abràmoff M, Folk J, Han D, Walker J, Williams D, Russell S, et al. Automated Analysis of Retinal Images for Detection of Referable Diabetic Retinopathy. *JAMA Ophthalmol* (2013) 131(3):351-7. doi: 10.1001/jamaophthalmol.2013.1743.
